# Next-Generation Site-Directed Transgenesis in the Malaria Vector Mosquito *Anopheles gambiae*: Self-Docking Strains Expressing Germline-Specific phiC31 Integrase

**DOI:** 10.1371/journal.pone.0059264

**Published:** 2013-03-13

**Authors:** Janet M. Meredith, Ann Underhill, Clare C. McArthur, Paul Eggleston

**Affiliations:** Centre for Applied Entomology and Parasitology, School of Life Sciences, Keele University, Keele, Staffordshire, United Kingdom; Centro de Pesquisas René Rachou, Brazil

## Abstract

Diseases transmitted by mosquitoes have a devastating impact on global health and the situation is complicated due to difficulties with both existing control measures and the impact of climate change. Genetically modified mosquitoes that are refractory to disease transmission are seen as having great potential in the delivery of novel control strategies. The *Streptomyces* phage phiC31 integrase system has been successfully adapted for site-directed transgene integration in a range of insects, thus overcoming many limitations due to size constraints and random integration associated with transposon-mediated transformation. Using this technology, we previously published the first site-directed transformation of *Anopheles gambiae*, the principal vector of human malaria. Mosquitoes were initially engineered to incorporate the phiC31 docking site at a defined genomic location. A second phase of genetic modification then achieved site-directed integration of an anti-malarial effector gene. In the current publication we report improved efficiency and utility of the phiC31 integrase system following the generation of *Anopheles gambiae* self-docking strains. Four independent strains, with docking sites at known locations on three different chromosome arms, were engineered to express integrase under control of the regulatory regions of the *nanos* gene from *Anopheles gambiae*. The resulting protein accumulates in the posterior oocyte to provide integrase activity at the site of germline development. Two self-docking strains, exhibiting significantly different levels of integrase expression, were assessed for site-directed transgene integration and found to demonstrate greatly improved survival and efficiency of transformation. In the fight against malaria, it is imperative to establish a broad repertoire of both anti-malarial effector genes and tissue-specific promoters to regulate their expression, enabling those offering maximum effect with minimum fitness cost to be identified. The improved technology we describe here will facilitate comparative studies of effector transgenes, allowing informed choices to be made that potentially lead to transmission blockade.

## Introduction

Despite intense efforts, malaria is globally responsible for almost one million deaths per year [1], the majority of which are in Africa. As a result of current interventions, the mosquito vectors are becoming increasingly resistant to pesticides [2] and the causative *Plasmodium* parasites are becoming similarly resistant to widely used anti-malarial drugs, including the most recent artemisinin-based combination therapies [3]. Faced with increased resistance and the lack of an effective vaccine, novel strategies are needed to complement the integrated pest management approach. Control measures that focus on the vector remain the most effective and deployment of transgenic mosquitoes that are refractory to malaria transmission is increasingly seen as having great potential [4]. This is particularly true in regions such as sub-Saharan Africa where transmission rates are very high and existing interventions are not expected to be sufficiently effective [5]. The focus of this study is therefore *An. gambiae*, the principal vector of malaria in endemic regions of Africa.

Transformation of several mosquito species has been achieved [6]–[12] and, in laboratory studies, some success has been reported in reducing the vectorial capacity of *An. stephensi*, the principal Asian malaria vector, to *Plasmodium* species [13]–[20]. Recently, we published the first report of an anti-malarial effector transgene in *An. gambiae* [21], which is a much more challenging organism for transgenic studies. However, there remains a dearth of information on both the activity of effector genes in *An. gambiae* and tissue-specific promoters that might be used to target *Plasmodium* parasites in this important vector insect [21], [22]. This imbalance between studies using transgenic *An. stephensi* compared to *An. gambiae* is in part due to the latter being a much more technically demanding model for transgene research. Significant advances are now required to enable both anti-malarial effector genes, and promoters for their controlled expression, to be compared directly and efficiently in transgenic *An. gambiae*. Such informed choices will raise the realistic prospect of transgenic approaches to the reduction of malaria transmission.

Historically, insect transgenesis has relied upon transposable genetic elements which, despite their utility, have limited carrying capacity and an essentially random integration profile that can cause insertional mutagenesis and position effects on transgene expression [23], [24]. *Streptomyces* phiC31 site-directed transgene integration circumvents these problems [25], [26]. The system can potentially accept much larger inserts than the 42.4 kb *Streptomyces* phage genome [27] and position effects due to transgene location are eliminated when comparing integrations into a previously characterised docking site. Site-specificity results from the two-phase nature of the transformation system. In phase 1, the phage attachment site (*attP*) is integrated into the genome using conventional transposon-mediated transgenesis. During phase 2 transformation, catalysed by phiC31 integrase, the *attP* site accepts transgenes from plasmids containing the bacterial attachment site (*attB*). Integration recombines the *attP* and *attB* sites into unique *attL* and *attR* sequences that are no longer recognised by the integrase, rendering insertions both unidirectional and stable.

We reported the first site-directed transformation of mosquitoes using the phiC31 system in the arboviral vector *Aedes aegypti* [26] and subsequently demonstrated its utility for expression of an anti-malarial effector transgene in *An. gambiae* [21]. Successful site-directed phiC31 integration has also been reported in *Ae. albopictus*, which is a vector for dengue and chikungunya [11]. Initial reports indicated substantially increased transformation efficiencies for phiC31 compared to transposon-mediated protocols in both *Drosophila melanogaster* and mosquitoes [26], [28]. However, subsequent studies reported variable efficiencies, suggesting that delivery of the integrase and, in particular, the quality and quantity of integrase mRNA injected into the embryos, may be problematic [11], [21], [29].

In *D. melanogaster* the phiC31 system was optimised by establishing an endogenous source of integrase [30] and we determined to transfer this improved technology to *An. gambiae*. Bischof *et al*. [30] compared the phiC31-mediated integration efficiencies of integrase expressed from the regulatory regions of both the *nanos* and *vasa* genes. The *nanos* control regions direct translation of maternal mRNA to the site of origin of the germ cells (reviewed in [31]), whilst *vasa* is responsible for directing the earliest zygotic transcription in germ cells [32]. In these experiments, transformation efficiencies into the most receptive docking site were increased by 100% when endogenous integrase was expressed using the *vasa* control regions. Use of a codon optimised integrase sequence (dphiC31), which differed at 172 nucleotides from the phage sequence, resulted in a further efficiency increase of almost 40% [30].

The results presented here demonstrate the transfer of an improved site-directed transformation system into *An. gambiae* by the generation of self-docking strains. These strains, containing *attP* docking sites at different chromosomal locations, use the regulatory regions of the *An. gambiae nanos* gene to direct a codon-optimised phiC31 integrase to the posterior oocyte, where the germline develops. We further show that two of these self-docking strains are amenable to site-directed transgene integration through uptake of an *attB*-containing reporter construct. These experiments resulted in increased post-injection survival and transformation efficiency compared to similar experiments based on the co-injection of *in vitro* transcribed integrase mRNA.

The availability of this simpler and more efficient transgenic protocol in *An. gambiae* will facilitate the characterisation and comparison of anti-malarial effector genes and the establishment of tissue-specific regulatory sequences for optimal expression of effector molecules in key mosquito tissues. It is anticipated that eliminating malaria transmission through transgenic mosquitoes and preventing evolution of parasite escape mechanisms will most likely require the expression of multiple transgenes in multiple tissues. The phiC31 system lends itself to the integration of such large, complex transgenes, having been shown to integrate BAC constructs of up to 133 kb [27]. Its use for the integration of complex constructs providing differential expression of multiple effector genes should be achievable in *An. gambiae* and will provide a valuable advance in the fight against malaria.

## Results

### Directing phiC31 integrase to the germline in *An. Gambiae*

**Figure 1:**
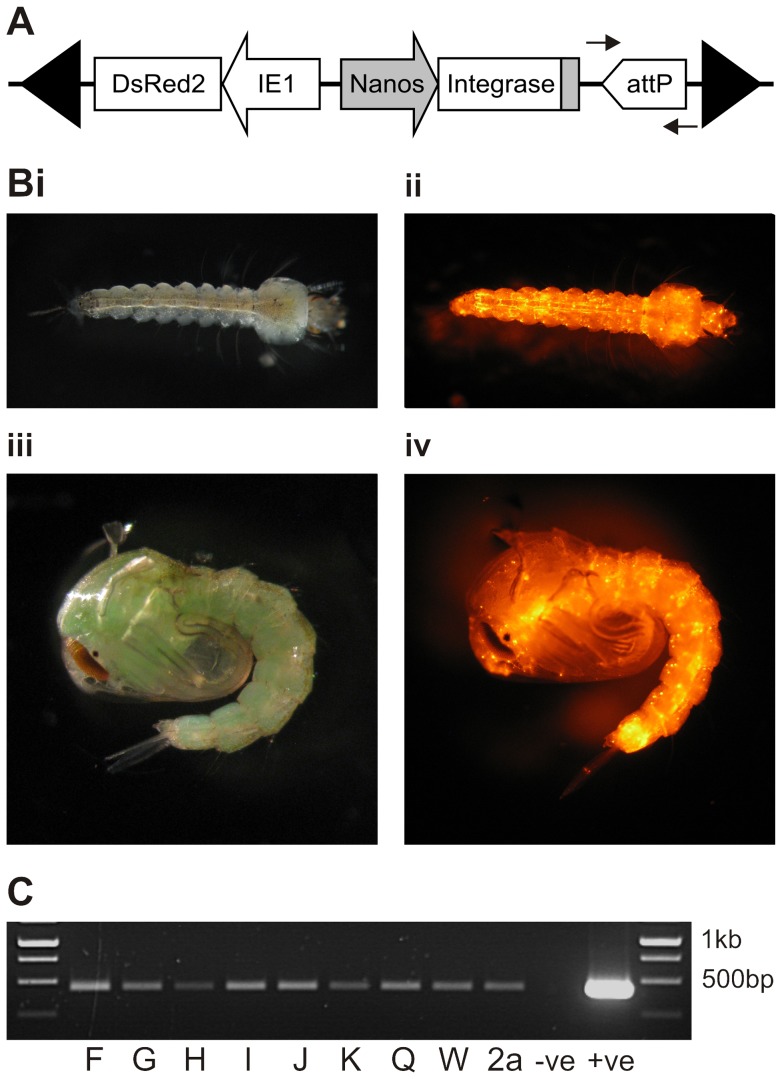
Generation of phase 1 self-docking strains. (A) Genomic organisation of the *piggyBac* insertion from pB*attP* [*nanos*-integrase]-[IE1-DsRed2nls] (not to scale) showing an unoccupied *attP* target site, integrase expressed from the *An. gambiae nanos* regulatory regions (promoter, 5′ and 3′UTRs in grey) and DsRed2 marker under control of the Hr5-IE1 promoter, all flanked by *piggyBac* left and right terminal inverted repeats (black triangles). Arrows represent the location of primers used to confirm presence of the *attP* docking site in genomic DNA. (B) Larvae and pupae of self-docking strain F. Panels (i) and (iii) are white light images, (ii) and (iv) are UV light DsRed2 fluorescence images of larvae (i and ii) or pupae (iii and iv). (C) PCR confirmation of *attP* integration (437 bp) from genomic DNA templates of phase 1 strains F, G, H, J, K, Q, W or 2a. The negative control lacks a template and the positive control is the phase 1 injection plasmid pB*attP* [*nanos*-integrase]-[hr5-IE1-DsRed2nls]. The marker is the GeneRuler 1 kb ladder (Fermentas).

The efficiency of phiC31-mediated transgene integration in *D. melanogaster* was significantly improved by directing endogenous integrase to the germline using either the *nanos* or *vasa* regulatory regions [30]. We pursued this objective in *An. gambiae* by expressing integrase from the regulatory regions of the *An. gambiae nanos* gene. The *An. gambiae* orthologue of the *D. melanogaster vasa* gene (AGAP008578 – VectorBase) was discounted when 5′-RACE identified two alternative splice variants in the 5′UTR (data not shown and GenBank accession number EU 522080) and subsequent characterisation implicated the 5′UTR sequences in sex-specific expression patterns [33]. In contrast, the *An. gambiae nanos* gene is not alternatively spliced [34]. We therefore designed a phase 1 plasmid to integrate the *attP* docking site into the genome and direct endogenous integrase expression to the germline (Figure 1A). In order to optimize integrase activity, we modified the coding sequence used in *Drosophila* [30] by incorporating synonymous codons (Figure S1) for arginine (CGC), leucine (CUG), serine (UCG), glycine (GGC) and valine (GUG) that are more commonly employed in *An. gambiae* [35]. The codon-optimized sequence was expressed from the regulatory regions of the *An. gambiae nanos* gene, comprising 1426 bp of upstream promoter region and both 5′ and 3′UTRs.

### Phase 1 transformation: generation of *An. gambiae* self-docking strains

2765 Keele embryos were co-injected with pB*attP* [*nanos*-integrase]-[hr5-IE1-DsRed2nls] (Figure 1A) and phsp-pBac with 21% surviving injection and 286 G_0_ adults backcrossed to Keele in 4 male and 4 female pools (Table 1). G_1_ progeny from multiple gonotrophic cycles were screened for DsRed2 and positive larvae were identified from two backcross pools. Fluorescence profiles of larvae and pupae were similar, with punctate distributions throughout the body (Figure 1B) and variable fluorescence intensities due to position effects. To establish strains arising from independent integration events, G_1_ adults exhibiting a range of DsRed2 intensities were separately backcrossed to Keele. From 29 backcrosses (11 G_1_ females and 18 G_1_ males) ten self-docking strains were established. Southern blot analysis (data not shown) revealed that two of these strains were identical and thus nine independent strains with single transgene insertions were taken forward for further analysis.

**Table 1:** Phase 1 injection statistics.

	embryos injected	G_0_ larvae hatched	G_0_ adults into backcrosses	independent transgenic strains
Phase 1	2765	573 (21%)	286	9

Survival and efficiency data following micro-injection of pB*attP* [*nanos*-integrase]-[hr5-IE1-DsRed2nls] (385 ng/µl) and phsp-pBac (224 ng/µl) into *An. gambiae* Keele strain embryos.

### Molecular characterisation of *An. gambiae* self-docking strains

**Figure 2:**
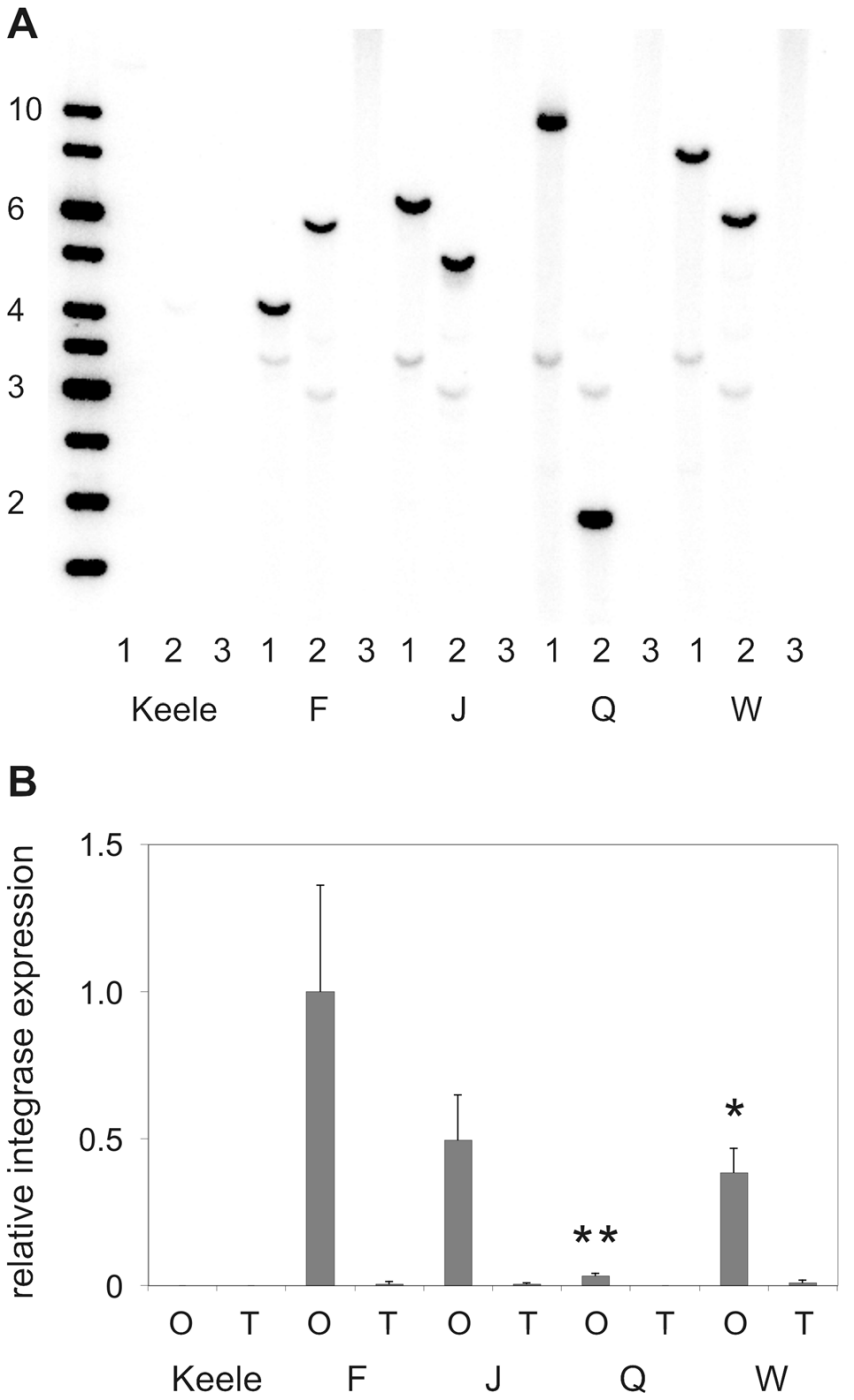
Characterisation of phase 1 self-docking strains. (A) Southern blot analysis of the wild-type Keele strain and transgenic self-docking strains F, J, Q and W. Genomic DNA was digested with *Pst*I (1), *Xmn*I (2) or uncut (3) and probed with a 741 bp *Mlu*I/*Nae*I fragment covering part of the *piggyBac* left terminal repeat. Single, unique hybridizing fragments are evident in each transgenic strain. Additional faint hybridization signals (approximately 3.5 kb and 3 kb following digestion with *Pst*I and *Xmn*I respectively) are absent in Keele DNA and independent of insertion site. These most likely result from weak homology between the probe sequence and the remainder of the inserted plasmid. Marker fragments (GeneRuler 1 kb) are shown to the left. (B) Real-time quantitative PCR analysis of integrase expression. The histogram shows relative expression of integrase from the *nanos* regulatory regions in the Keele strain and transgenic strains F, J, Q and W. Samples were collected from ovaries (O) and thoraces (T) 72 hrs post-bloodmeal. Integrase expression levels were normalised to S7 and means with standard deviations from 3 independent experiments plotted relative to expression in strain F ovaries. Significantly different ovarian expression levels are shown relative to F for strains Q (*P* = 0.002; **) and W (*P* = 0.033; **).

The presence of canonical *attP* sequences was confirmed in all 9 strains by amplification and sequencing of the region spanning *attP* from genomic DNA (Figure 1C). The relative abundance of integrase transcripts, investigated by semi-quantitative RT-PCR, was highly variable due to position effects (Figure S2). Inverse PCR was used to identify transgene insertion sites in six strains (Table 2). Four strains (F, J, Q, W) were retained for further analysis and the presence of single-copy, unique transgene insertions confirmed by Southern blotting (Figure 2A). Relative levels of integrase expression in these strains were determined by real-time quantitative PCR on total RNA extracted from ovaries 72 hrs post blood-meal (Figure 2B). Pairwise comparisons identified significantly different levels of integrase expression between strain F and strains Q (*P* = 0.002) and W (*P* = 0.033) respectively.

**Table 2:** Location of phase 1 *attP* integrations.

strain	chromosomal location	insertion site	nearest predicted gene
F	2L 23C	23661650	AGAP005918 (novel protein)
			insert located in intron within 5′UTR
J	3R 36B	46699965	AGAP009964 (novel protein)
			insert located 2kb downstream
K	3L 40A	11349775	AGAP010822 (novel protein)
			insert located 6.9kb upstream
Q	3L 41A	15200903	AGAP011034 (known protein coding)
			insert located 98kb downstream
W	3L 43A	24273635	AGAP011419 (known protein coding)
			insert located 57kb upstream
2a	3L 45A	35935122	AGAP011990 candidate odorant receptor
			insert located within coding region

For each strain, the chromosomal location (number, arm and polytene map division), is shown, followed by the nucleotide number of the insertion site on the reference PEST scaffolds. Also shown is the location of the nearest predicted gene within the PEST genome assembly. All insertion sites are unique and into canonical *piggyBac* TTAA sequence motifs.

### Phase 2 transformation: site-directed transgene integration

**Figure 3:**
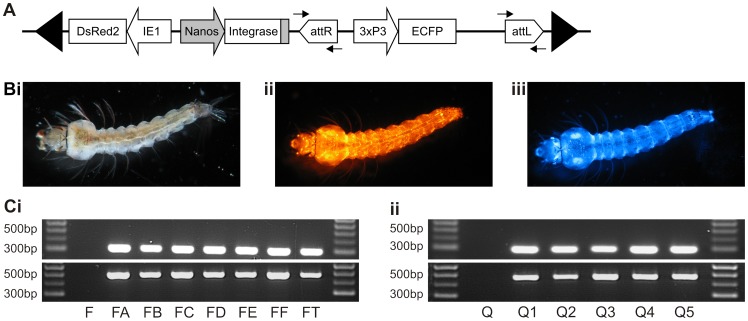
Confirmation of phase 2 site-directed transgene integration. (A) Genomic organisation following site-directed integration of pB*attB* [3xP3-ECFP] into a phase 1 *attP* site (not to scale). Arrows represent the location of primers used to amplify *attL* and *attR* following transgene uptake. (B) Images of a strain FA larva under (i) white light, (ii) UV light with red filter set showing hr5-IE1 DsRed2 fluorescence profile and (iii) UV light with blue filter set showing 3xP3 ECFP fluorescence profile after phase 2 transgene uptake. (C) PCR confirmation of the presence of *attL* (301 bp, upper panels) and *attR* (485 bp, lower panels) motifs in genomic DNA from (i) phase 2 strains FA, FB, FC, FD, FE, FF and FT derived from self-docking strain F and (ii) phase 2 strains Q1, Q2, Q3, Q4 and Q5 derived from self-docking strain Q. The marker ladder is Hyperladder IV (Bioline).

Since the level of integrase expression required for successful transgene uptake was unknown, we investigated the ability of two self-docking strains with significantly different expression levels to integrate the cyan fluorescence reporter pB*attB* [3xP3-ECFP]. Site-directed uptake of this reporter results in the genomic organization shown in Figure 3A. For strain F, 2308 embryos were injected and 60% hatched (Table 3). Of these, 30% were negative for DsRed2 and discarded. Following adult eclosion, 423 G_0_ survivors were backcrossed to Keele in six male and five female pools and G_1_ progeny screened for ECFP. Multiple positive G_1_ larvae were identified in seven of the 11 backcross populations (Figure 3B). For strain Q, 1358 embryos were injected and 55% survived (Table 3). Of these, 273 G_0_ adult survivors were positive for DsRed2 expression and were backcrossed to Keele in six female and ten male pools. Five of the 13 surviving pools yielded ECFP-positive G_1_ progeny. In all cases, site-directed integration into the docking site was confirmed by PCR analysis of genomic DNA and sequencing of the resulting products (Figure 3C). These data confirm that both self-docking strains are amenable to site-directed transgene integration and indicate that the amount of integrase mRNA transcribed is not limiting.

**Table 3:** Phase 2 injection statistics.

	embryos injected	G_0_ larvae hatched	G_0_ adults in backcrosses	independent transgenic strains
Phase 2 into F	2308	1374 (60%)	423	7
Phase 2 into Q	1358	750 (55%)	280	5

Survival and efficiency data following micro-injection of pB*attB* [3xP3-ECFP] (300 ng/µl) into *An. gambiae* self-docking strains F and Q.

## Discussion

The application of recombinase-mediated transgenic technologies and, in particular, use of the uni-directional phiC31 integrase, has proven very effective in insect genetic modification. This technology has now been established in *Drosophila* [28], *Aedes aegypti* [26], *Ceratitis capitata* [36], *Aedes albopictus* [11], *Anopheles gambiae* [21] and *Anopheles stephensi* [18]. The ability to direct transgenes to a specific docking site within the genome offers distinct advantages over the use of transposable elements. Most importantly, it provides a mechanism for *in vivo* comparisons of effector genes and regulatory sequences in identical genomic environments. It also offers higher transformation efficiencies, circumvents the potential for position effects or insertional mutagenesis and offers greater carrying capacity [37]. Indeed, phiC31 has been used successfully to integrate BAC vectors up to 133 kb in *D. melanogaster*, even though some decline in efficiency was noted above 50 kb [27]. Use of this system will therefore facilitate the incorporation of large and complex constructs expressing multiple effector genes into the same or different tissues.

Outside of *Drosophila*, where the technical demands of insect transgenesis are greater, there have been indications that successful delivery of phiC31, either using helper plasmids or *in vitro* transcribed mRNA, can be problematic. In both cases, the high concentrations of nucleic acid required can negatively impact embryo survival and the quality and functionality of synthesized mRNA is critical. In *Drosophila*, these limitations were addressed by creating strains that expressed their own integrase, resulting in significant efficiency gains [30]. In this study we generated a number of phiC31 self-docking strains, designed to advance transgenic technologies in *An. gambiae*, which express endogenous integrase in the posterior oocyte at the site of germline development. Integrase expression was directed to this region using the *An. gambiae nanos* regulatory regions and functionality was confirmed by site-directed integration of a fluorescence reporter gene in two independent strains.

In our experiments, we noted significant improvements in embryo survival during phase 2 injections. Strains F and Q yielded average survival rates of 59% and 55% respectively, compared to typical rates of 20% for standard protocols. This provides an immediate efficiency gain through reductions in the number of embryos that need to be injected for transformation. We suggest that the increased survival is due to the lower concentrations and viscosities of injected nucleic acid. The increased survival rate also translates into improved transformation efficiencies relative to embryos injected. For strains F and Q, we generated a minimum of seven and five independent integration events respectively. However, since all phase 2 integrations are phenotypically identical, G_0_ adults are pooled and some backcrosses generate large numbers of positive G_1_ larvae. Thus, the actual number of independent integration events could well be higher.

Comparisons of transformation efficiencies are notoriously difficult since they are dependent on approach and husbandry techniques. Franz *et al*. [29] reported very similar or lower transformation efficiencies with transposable elements compared to integrase in *Ae. aegypti*. These workers also tried unsuccessfully to improve the phiC31 integration efficiencies by injecting integrase helper plasmid rather than *in vitro* transcribed mRNA. Labbé *et al*. [11] compared the two systems in *Ae. albopictus* and reported very similar transformation efficiencies. In these experiments, transformation efficiencies were expressed relative to the number of fertile injection survivors going into backcrosses, based on a 50% sterility rate reported for G_0_ *Ae. aegypti* following microinjection [38]. Similar information is not available for *An. gambiae*, nor would single adult crosses be feasible. In fact, numbers of adults in each backcross were kept high (at the risk of missing independent events) in order to optimise the number G_1_ progeny recovered. The transformation efficiencies that we report here for *An. gambiae* are therefore likely to be underestimates.

Microinjections into self-docking strains F and Q used populations that were enriched, but not homozygous, for the *attP* docking site. Injected embryos were therefore either hemizygous or homozygous for the docking site. Moreover, real-time qPCR data indicates that integrase expression levels are not limiting (Table 3). Thus, in addition to their use as described here, our strains could also be used as integrase ‘driver’ lines to increase the range and distribution of available self-docking sites. For example, strain Q homozygotes could be crossed to a conventional docking strain to yield embryos hemizygous for two independent docking sites that also express germline-specific phiC31 integrase. This is an important consideration given the known variability in receptivity identified in *Drosophila* [27].

Mutational derivatives of phiC31 integrase have been reported to have increased efficiency and specificity in cultured human cells [39]. However, in *Ae. aegypti* and *An. stephensi*, transformation efficiencies using the P3 variant were no higher than those with wild-type integrase [18], [29]. The P3 integrase includes nine amino acid substitutions and an additional 33 amino acid N-terminal extension and these experiments used *in vitro* transcribed mRNA, codon optimised for expression in *An. stephensi* [40]. In our experiments, we chose to base our integrase expression on the dphiC31 coding sequence [30] and made further nucleotide substitutions to account for *An. gambiae* codon usage [35]. The success we obtained from very low levels of integrase expression would suggest that further variations of the phiC31 integrase are unnecessary in *An. gambiae*.

Further refinements to the phiC31 system for use in *Anopheles* could include stabilisation of the initial *piggyBac* insertion by removal of one or both termini [36], [41], [42]. This may, in fact, be a regulatory requirement for insects that are destined for release into the wild. Insects appear to differ in their potential for remobilisation of *piggyBac*. In *Ae. aegypti*, no evidence for either somatic or germline remobilization has been found [43]. However, *piggyBac* shows high levels of germline remobilization in *An. stephensi* [44], *D. melanogaster* [45] and *Tribolium castaneum* [46]. Should potential re-mobilisation in *An. gambiae* be seen as an issue with respect to either phenotypic stability or regulatory approval, driver strains designed to stabilize the docking site could be deployed. Such an integration and stabilisation system was designed using the phiC31 system in the Mediterranean fruit fly, *Ceratitis capitata* [36]. An advantage of the self-docking system is that stabilization would only need to be done once, rather than on each newly derived transgenic strain. Other options to be considered for future docking strains would be removal of the entire integrase expression cassette, together with its associated fluorescent marker, once site-directed transgene integration had been accomplished. Cre-*loxP* excision was previously shown to be highly efficient for removal of a marker from transgenic *Ae. aegypti* [47]. Excision could therefore be accomplished by the inclusion of *loxP* sites either side of the phase 1 integrase and marker cassettes.

In addition to the improvements to transformation technology outlined here, site-directed transgene integration also opens up new areas of research. Windbichler *et al*. recently published a description of a homing endonuclease-based gene drive system that would not have been possible without docking systems [48]. Using a characterised *attP* docking strain [21], they were able to insert donor, target and reporter constructs into the same chromosomal location to facilitate ‘homing’ by recombinational repair. Nolan *et al*. also advocated the use of phiC31 for the construction of genetic sexing strains [49] since transposition events on the Y-chromosome are extremely rare. Once generated, such a docking strain would allow for the subsequent insertion and comparison of potential sexing strains.

The work we present here shows how next-generation transgenesis techniques can be extended to non-model insects beyond *D. melanogaster*. We believe that they highlight the critical issues that need to be addressed and provide a platform for the broader development of such technologies in many insects of medical and/or economic importance.

## Materials and Methods

### Plasmids

The phase 1 construct, pB*attP* [*nanos*-integrase]-[hr5-IE1-DsRed2nls], was constructed based on the previously described construct pBac [3xP3-ECFPaf]-*attP* [26]. Firstly the plasmid was simplified by removal of a polylinker sequence (nucleotides 2233 to 2266). Following digestion with *Sal*I (partial) and *Eco*RI, ends were filled in with the Klenow fragment of DNA polymerase I (Promega) and self-ligated (T4 DNA ligase, Promega) to generate pBac [3xP3-ECFPaf]-*attP*-Pd. The fluorescent marker cassette, hr5-IE1-scraps-DsRed2-SV40 was excised using unique *Kpn*I/*Hpa*I sites from plasmid 923 (Oxitec Ltd.) and cloned into similarly cut pBac [3xP3-ECFPaf]-*attP*-Pd to replace the ECFP marker up to the *Hpa*I site, located internally within the SV40 polyadenylation signal of both markers, thus generating pB*attP* [hr5-IE1-DsRed2nls]-Pd. Plasmid 923 was used solely as a source of a standard reporter cassette with widely used components. The reporter is the DsRed2 fluorophore and this is expressed from the *Autographa californica* nuclear polyhedrosis virus (baculovirus) immediate early gene 1 promoter (IE1) linked to the *Autographa californica* nuclear polyhedrosis virus homologous region 5 (hr5) enhancer. An intron from the *D. melanogaster* scraps (anillin) gene is located between the promoter and reporter to optimise expression and transcription is terminated by the standard SV40 polyadenylation signal. The baculovirus IE1 promoter is widely used in insect gene expression studies and is a constitutive promoter that gives high levels of expression in all tissues. The integrase cassette was synthesised by GenScript Corporation, NJ, USA and supplied cloned into pUC57. The design for the integrase coding region was based on that published for use in *D. melanogaster* [30]. We chose to optimise codon usage for *An. gambiae* by changing codons that occurred in less than 10% of cases in an earlier study [35] to more favourable codons (Figure S1). In addition, a PKKKRKV nuclear localisation sequence, used for *Drosophila* expression [30], was added immediately before the stop codon. The entire sequence was flanked by the *An. gambiae nanos* UTR sequences (Figure S1), designed based on the PEST genome sequence, with 2 base changes and 7 additions in the 3′UTR to match both the published transcript (AY583530) [34] and 3′-RACE performed on the Keele strain (data not shown). The *nanos* non-coding sequences comprised the last 13 nucleotides of the 5′UTR (from the *Xba*I site) and 642 bp of downstream sequence to include the 3′UTR (304 bp). Finally a 3′ *Bam*HI site was added to facilitate subsequent cloning experiments. The remainder of the *nanos* 5′UTR, together with 1426 bp of upstream promoter sequence, was amplified from KIL genomic DNA using the primers [*nanos* prom fwd], which also added a 5′ *Kpn*I site, and [*nanos* prom rev] both designed against the PEST genome sequence. Following sequence confirmation the PCR fragment was cloned upstream of the integrase coding region using the introduced *Kpn*I site and the *Xba*I site within the *nanos* 5′UTR of both the amplified and synthesised fragments. The final plasmid was completed by transferring the integrase expression cassette into pB*attP* [hr5-IE1-DsRed2nls]-Pd using the unique *Kpn*I and *Bam*HI sites, which removed the redundant 3xP3 promoter, to generate pB*attP* [*nanos*-integrase]-[hr5-IE1-DsRed2nls]. All relevant primer sequences are given in Table S1. The transposase helper plasmid phsp-pBac has been described previously [50]. The phase 2 transformation plasmid pB*attB* [3xP3-ECFP] was constructed by transferring the *Apa*I *attB* fragment from pBCPB + [28] into *Apa*I cut pBluescript II to generate pBluescriptII-*attB*. The 3xP3-ECFP fluorescent marker from pBac [3xP3-ECFPaf]-*attP* [26] was introduced on an *Avr*II-*Pst*I fragment by ligating into pBluescriptII-*attB* which was cut with *Spe*I and *Pst*I. Microinjection DNA was prepared using the EndoFree Plasmid Maxi kit (Qiagen).

#### Insect strains

*An. gambiae* (Keele) and all transgenic derivatives were maintained at 26°C±1°C and 80% RH in a 12-hour light: 12-hour dark photoperiod. Larvae were reared under standardised conditions [51] and adults fed 10% glucose *ad libitum*. Females (3–5 days old) were blood fed and pre-blastoderm embryos collected for microinjection 48–96 hours post-bloodmeal.

### Microinjection

Microinjection was performed as previously reported [26] except that embryos were not recovered onto filter paper. For phase 1 experiments, wild-type Keele strain embryos were co-injected with pB*attP* [*nanos*-integrase]-[hr5-IE1-DsRed2nls] at 385 ng/µl and phsp-pBac at 224 ng/µl in 1× injection buffer and recovered without heat shock. For phase 2, strain F or strain Q embryos were injected with pB*attB* [3xP3-ECFP] at 300 ng/μl. Surviving G_0_ males were backcrossed to a 4 or 5-fold excess of Keele females in pools of 4–10 to optimise mating opportunities for putative transformants. G_0_ females were backcrossed in pools based on day of emergence. Putative G_1_ transformants were identified by screening for fluorescence (Leica MZ FLIII) with filter sets from Chroma Technology (ECFP: exciter D436/20x; emitter D480/40m; DsRed: exciter HQ545/30x; emitter HQ620/60m). Transgenic strains were established from single G_1_ positive adults by backcrossing to Keele. White light and fluorescence images were taken using a Canon PowerShot S518 camera with MM99 adaptor (Martin Microscope Company).

### Southern blotting

Genomic DNA was isolated using the Puregene DNA Purification Kit (Gentra systems) from 10 headless mosquitoes, crushed using Pellet Pestles (Anachem Ltd), in a 5× modification of the manufacturer's *Drosophila* genomic purification protocol and re-suspended in a final volume of 50 µl. Approximately 15 µg of genomic DNA was digested with *Xmn*I or *Pst*I, separated on 0.7% agarose and blotted onto Hybond-N+ (Amersham Biosciences). The 741 bp probe used to detect *piggyBac* integrations was generated by *Mlu*I/*Nae*I digestion of pB*attP* [hr5-IE1-DsRed2nls]-Pd. This fragment, covering a portion of the *piggyBac* left terminus, was labelled with [α-^32^P]dCTP using Ready-To-Go DNA labelling beads (Amersham Biosciences). Blots were exposed to phosphor screens and scanned (Cyclone Storage Phosphor Screen, Packard BioScience). Fragment sizes were determined by comparison to the GeneRuler 1 kb DNA ladder (Fermentas).

#### Inverse PCR

Inverse PCR was performed as described previously [26]. For strains F and Q the 5′ junction sequence was obtained using *Hae*III digestion and primers [5′FOR] and [5′REV]. For strains J, K, W and 2a, the 3′ junctions were resolved using *Hae*III digestion together with primers [3′FORnew] and [3′REVnew]. PCR products were purified (Wizard SV Gel and PCR Clean-Up system, Promega) prior to sequencing (Eurofins MWG Operon, Germany) with the corresponding reverse primer for either 5′ or 3′ junctions. Primer sequences are given in Table S1.

### PCR analysis of site-directed integration

Unoccupied *attP* docking sites were identified using primers [*attP*-int-fwd] with [*attP*-int-rev]. Occupied docking sites were identified using primers to amplify *attL* [*attL*-F-new-2] with [*attL*-R-new-2] and *attR* [*attR*-F-new2] with [*attR*-R-new2]. Genomic DNA (250 ng) was amplified with the relevant primers (0.4 µM) using DNA polymerase (GoTaq, Promega) in 1× buffer containing 1 mM MgCl_2_. Cycling parameters (MJ Research PTC-100) were 94°C for 1 minute then 30 cycles (94°C, 30 seconds; 53°C (*attP*), 54°C (*attL*) or 50°C (*attR*), 30 seconds; 72°C, 45 seconds) followed by a final extension at 72°C for 10 minutes. PCR products were separated on 1.5% agarose with *attP* visualized against the GeneRuler 1 kb DNA ladder (Fermentas) and *attL or attR* against HyperLadder IV (Bioline). PCR products were purified (Wizard SV Gel and PCR Clean-Up system, Promega) prior to sequencing (Eurofins, Germany) with either the forward primer (*attP*) or the reverse primer (*attL* and *attR*). Primer sequences are given in Table S1.

### RT-PCR and real-time quantitative PCR

Female mosquitoes (3 to 4 days old) were bloodfed and collected on ice 72 hours post-bloodmeal for dissection. Total RNA was extracted from ovaries (RNeasy Plus Mini kit, Qiagen) or thoraces (TRIzol, Invitrogen). For RT-PCR, cDNA synthesis used random primers with 5 µg RNA and Superscript III (Invitrogen). To amplify the integrase transcript we used primers *nanos*-int-fwd with *nanos*-int-rev. To amplify rpL7a control transcripts we used primers rpLfwd with rpLrev to (Table S1). By limiting cycle numbers to the exponential phase (20 for rpL7a and 25 for *nanos* integrase) reactions were semi-quantitative and band intensity could be quantified (Bio Imaging Systems, Syngene Europe). Cycling parameters were as described above for PCR analysis, using 10% of the RT reaction as template with annealing at 54°C (*nanos*-int) or 57°C (rpL7a), except that extension was for 1 min. For real-time quantitative PCR, we carried out three independent experiments, each using ovaries and thoraces from 30 females, collected separately and frozen at −80°C. For cDNA synthesis we used random primers with 2.5 µg RNA and the Superscript VILO cDNA Synthesis Kit (Invitrogen). Subsequently, 2 µl of a 1∶2 dilution of cDNA was used with Power SYBR;Green PCR (Applied Biosystems) for PCR. Primers to amplify *nanos* integrase and S7 were [*nanos*intqPCRfwd] with [*nanos*intqPCRrev] (both at 50 nM) and [S7qPCRfwd] with [newS7qPCRrev] (both at 300 nM) respectively. Reactions used the standard amplification protocol (ABI Prism 7000 sequence detection system, Applied Biosystems), including a dissociation protocol, and were run in duplicate with 3 biological replicates. Relative quantitation of gene expression followed the standard curve method in User Bulletin #2 (Applied Biosystems). Standard curves for *nanos*: integrase or S7 were generated from 10-fold dilutions (1 ng to 1fg) of pB*attP* [*nanos*-integrase]-[hr5-IE1-DsRed2nls] or an S7 368 bp PCR fragment, amplified from Keele strain total RNA using [S7fwd] with [S7rev] (Table S1) and cloned into the TOPO 2.1 vector (Invitrogen) respectively. Following normalization to S7, integrase expression data from 3 experiments, with 2 replicates in each, were pooled for analysis. Log-transformed data, checked for normal distribution, were analysed by ANOVA (General Linear Model) with Tukey's pairwise comparisons using MINITAB.

## Supporting Information

Figure S1 **Synthesized DNA fragment for codon-optimized expression of phiC31 integrase in** ***Anopheles gambiae*** **.** The integrase coding sequence (triplet codons) is flanked by the final 13 nucleotides of the 5′UTR and 642 nucleotides of 3′ sequence including the 3′UTR from the *Anopheles gambiae nanos* gene, both shown as continuous sequence. Synonymous codon changes from the dphiC31 *Drosophila* optimised coding sequence [1] are highlighted as follows, with the number of changes given in brackets:- arginine agg to cgc, shown in yellow (7), leucine uug or cuu to cug, shown in green (18), serine uca to ucg, shown in light blue (2), glycine ggg to ggc, shown in magenta (2) and valine gua to gug, shown in grey (1). Additionally, asparagine gac to gat at nucleotide 19 (shown in red) generated a *Sna*BI site (tacgta), which together with a *Tth*111I site (gacagagtc) immediately following the stop codon (tag), allows for expression of alternative peptides from the *nanos* control regions. A nuclear localisation signal (proline, lysine, lysine, lysine, arginine, lysine, valine – shown in bold capitals), was added immediately before the stop codon. Changes to the PEST reference genome 3′UTR sequence (2 base changes and 7 additional bases) are shown in dark blue. The entire fragment is flanked by an *Xba*I site (tctaga) in the 5′UTR and a *Bam*HI site (ggatcc) added to the 3′ end (both underlined).1. Bischof J, Maeda RK, Hediger M, Karch F, Basler K (2007) An optimized transgenesis system for *Drosophila* using germ-line-specific phiC31 integrases. Proc Natl Acad Sci U S A 104: 3312–3317.
(DOC)

Figure S2 **Semi-quantitative RT-PCR of relative integrase expression levels.** The histogram shows relative expression of phiC31 integrase at 72 hrs post blood-meal in the wild type Keele strain and nine independent transgenic strains engineered to express integrase (F, G, H, I, J, K, Q, W and 2a). All expression levels were normalized to strain F, which gave the highest levels of integrase expression.
(TIF)

Table S1 (DOC)
