# Supplementary material for: Next-Generation Site-Directed Transgenesis in the Malaria Vector Mosquito Anopheles gambiae: Self-Docking Strains Expressing Germline-Specific phiC31 Integrase
Source: PLoS One. 2013 Mar 13;8(3):e59264. doi: 10.1371/journal.pone.0059264 (PMC3596282; doi:10.1371/journal.pone.0059264)
Supplement: Figure S1 — Synthesized DNA fragment for codon-optimized expression of phiC31 integrase in Anopheles gambiae . The integrase coding sequence (triplet codons) is flanked by the final 13 nucleotides of the 5′UTR and 642 nucleotides of 3′ sequence including the 3′UTR from the Anopheles gambiae nanos gene, both shown as continuous sequence. Synonymous codon changes from the dphiC31 Drosophila optimised coding sequence [1] are highlighted as follows, with the number of changes given in brackets:- arginine agg to cgc, shown in yellow (7), leucine uug or cuu to cug, shown in green (18), serine uca to ucg, shown in light blue (2), glycine ggg to ggc, shown in magenta (2) and valine gua to gug, shown in grey (1). Additionally, asparagine gac to gat at nucleotide 19 (shown in red) generated a SnaBI site (tacgta), which together with a Tth111I site (gacagagtc) immediately following the stop codon (tag), allows for expression of alternative peptides from the nanos control regions. A nuclear localisation signal (proline, lysine, lysine, lysine, arginine, lysine, valine – shown in bold capitals), was added immediately before the stop codon. Changes to the PEST reference genome 3′UTR sequence (2 base changes and 7 additional bases) are shown in dark blue. The entire fragment is flanked by an XbaI site (tctaga) in the 5′UTR and a BamHI site (ggatcc) added to the 3′ end (both underlined).1. Bischof J, Maeda RK, Hediger M, Karch F, Basler K (2007) An optimized transgenesis system for Drosophila using germ-line-specific phiC31 integrases. Proc Natl Acad Sci U S A 104: 3312–3317. (DOC) [file pone.0059264.s001.doc]

tctagaaagcaag atg gat acg tat gcc ggt gct tac gac cgt cag agc cgc gag cgg gaa aat tcc agc gca gca agt cca gcc aca cag cgt agc gcc aac gag gac aag gcg gcc gac ctg cag cga gaa gtc gag cga gat ggt ggc cgc ttc cgc ttc gtc ggc cat ttc tcc gaa gct cca ggc act agc gcg ttc gga aca gcc gag cgc ccg gag ttc gaa cgc atc ctg aac gag tgc cgt gcc ggc cgc ctc aac atg atc ata gtc tat gac gtg agt cgc ttc tcg cgc ctg aag gtc atg gac gcg att ccg att gtg agc gaa ctg ctc gcc ctg ggt gtg acg ata gtg tcc acg cag gaa ggc gtg ttt cgg cag gga aac gtc atg gac ctg ata cac ctg att atg cgc ctg gac gct tcg cac aag gag tcc agc ctg aag agt gcg aag att ctc gac acc aag aac ctg cag cgc gaa ctg ggc ggt tat gtc ggc ggt aag gct ccc tac ggc ttc gag ctg gtt tcg gag acg aag gag atc acg cgt aac ggc cga atg gtc aat gtc gtg atc aac aag ctg gcc cac tcg acc aca ccc ctg acc gga ccc ttc gag ttc gag ccc gac gtt atc cgt tgg tgg tgg cgt gag atc aag acc cac aag cac ctg ccc ttc aag cca ggc agt caa gcc gcc att cac ccg ggc agc att acg ggc ctg tgt aag cgc atg gac gca gat gct gtg cca aca cgc ggt gag acg att ggc aag aag acc gct tcg agc gcc tgg gac ccc gca acc gtt atg cga atc ctg cgt gac ccg cgt att gca ggc ttt gcc gct gag gtg atc tac aag aag aag ccg gat gga acg ccg acc acg aag ata gag ggt tac cgc att cag cgc gat ccg atc acc ctg cgc ccc gtg gag ctc gat tgc gga ccc atc atc gag ccc gct gag tgg tat gag ctg cag gcg tgg ctg gat ggc cgc ggt cgc ggc aag ggt ctg tcc cgg gga caa gcc ata ctg agc gcc atg gac aag ctg tac tgc gaa tgt ggc gcc gtg atg act tcg aag cgc ggc gag gaa tcg atc aag gac tcc tac cgc tgc cgt cga cgg aag gtg gtt gac cca agc gca cct ggt cag cat gag ggc acg tgc aac gtg agc atg gcg gca ctc gac aag ttc gtt gcg gaa cgc atc ttc aac aag atc cgc cac gct gaa ggc gac gaa gag acg ttg gcc ctg ctg tgg gaa gcc gcg cga cgc ttc ggc aag ctc act gag gcg cca gag aag tcc ggc gaa cgg gcg aac ctg gtt gcg gag cga gcc gat gcc ctg aac gcc ctc gaa gag ctg tac gaa gac cgc gct gca gga gct tac gac gga ccc gtt ggc cgc aag cac ttc cgg aag caa cag gca gcg ctg aca ctc cgg cag caa gga gca gaa gag cga ctg gcc gag ctg gaa gcc gcc gaa gcc cca aag ctc ccc ctg gac caa tgg ttc ccc gaa gac gcc gac gct gac cca act gga ccg aag tcg tgg tgg gga cgc gca tcg gtg gac gac aag cgc gtg ttt gtg ggc ctc ttc gtg gac aag atc gtt gtg acc aag agc act acc ggc agg gga cag gga aca ccc atc gag aag cgc gct tcg atc acg tgg gca aag ccc ccg acc gat gac gat gaa gat gac gcc cag gat ggc acc gaa gac gtg gcg gcc **CCG AAG AAG AAG CGC AAG GTG** tag gacagagtcgttcgttcattccttttttattactttacaacacatcc

aaagctctgtgagcttcaagcaacaggtagtagctgacatcggaactggtgggcaagaaaggcttgc

agcaaata**c**gtttttggctgctc**a**gagaatgt**gaagctt**gaagatatatttatttaggaaaagtgga

actttatgcaggatgaataattttgccatcgaatcaaatagcgtaagtaggtagagtgaaaaatcga

tcttaaaaggatgatttccacgttcgaacattacacattaaggatggtatccatacatacgaatgcg

gtttaaattcaatatttaccttgaagcagatgttcgtatcatttcctccttagcatctttatgtcta

tacttcttttaaggacaacatttatagattttttgatacaacgaatcattttctatgaatcaatcat

ttgatgcattgattaaataaattgcgaagaaatatttaacaacaatcgattctaaatgtgttgttag

aaggtacaaccaaaaaacctttaatgtcttggagcgaatgttcaaagatattgcttagcctctctct

tcggctaaaatgaacactaattaccataacttcgtataatgtatgctatacgaagttatggatcc
