# Supplementary figures and images for: Next-Generation Site-Directed Transgenesis in the Malaria Vector Mosquito Anopheles gambiae: Self-Docking Strains Expressing Germline-Specific phiC31 Integrase
Source: PLoS One. 2013 Mar 13;8(3):e59264. doi: 10.1371/journal.pone.0059264 (PMC3596282; doi:10.1371/journal.pone.0059264)

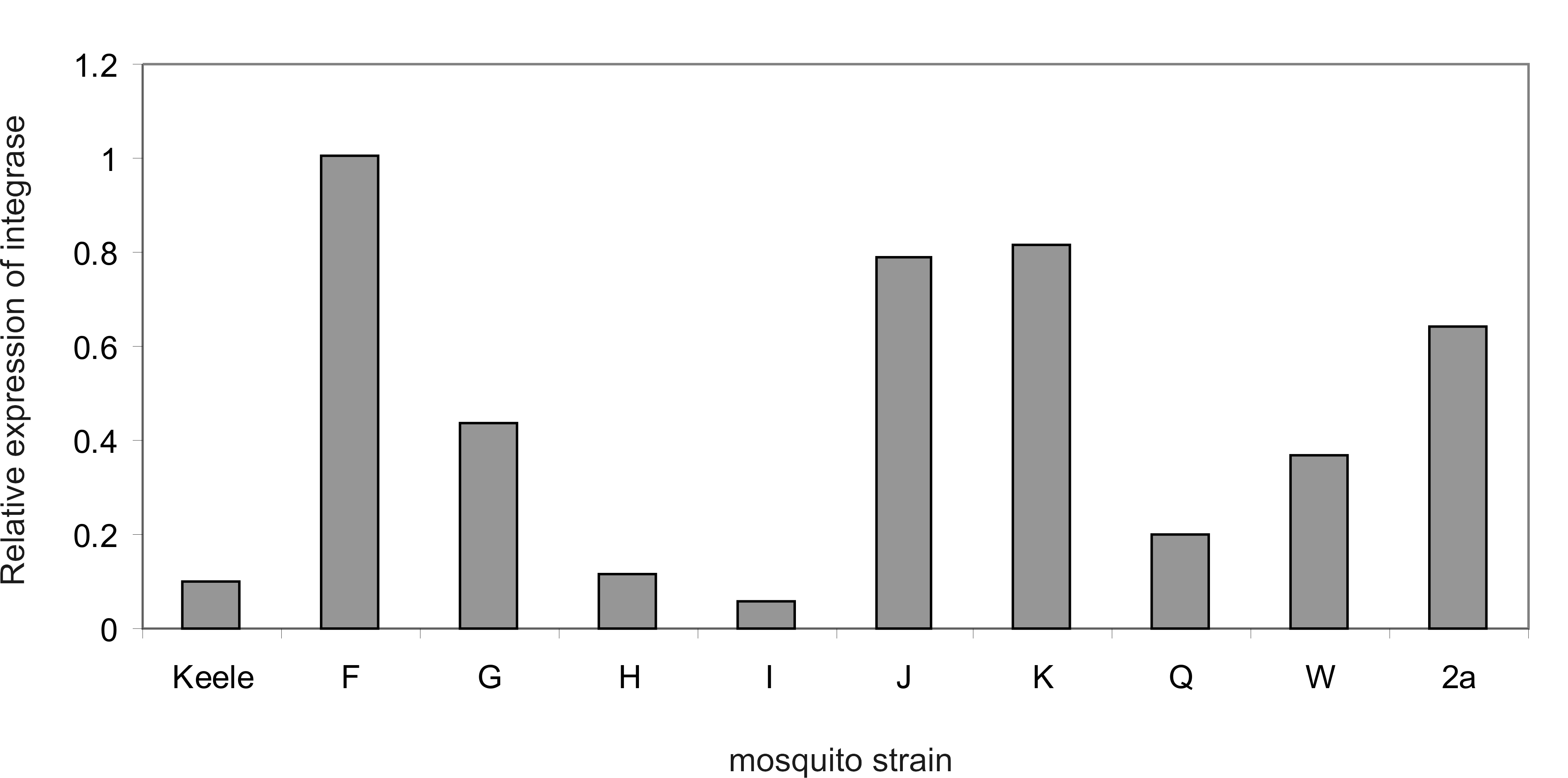

Supplement: Figure S2 — Semi-quantitative RT-PCR of relative integrase expression levels. The histogram shows relative expression of phiC31 integrase at 72 hrs post blood-meal in the wild type Keele strain and nine independent transgenic strains engineered to express integrase (F, G, H, I, J, K, Q, W and 2a). All expression levels were normalized to strain F, which gave the highest levels of integrase expression. (TIF) [file pone.0059264.s002.tif]
