# Supplementary material for: Next-Generation Site-Directed Transgenesis in the Malaria Vector Mosquito Anopheles gambiae: Self-Docking Strains Expressing Germline-Specific phiC31 Integrase
Source: PLoS One. 2013 Mar 13;8(3):e59264. doi: 10.1371/journal.pone.0059264 (PMC3596282; doi:10.1371/journal.pone.0059264)
Supplement: Table S1 — (DOC) [file pone.0059264.s003.doc]

**Table S1. Primers.**

| **Oligonucleotide** | **Sequence** |
| --- | --- |
| *nanos* prom fwd | 5’-ACTGGTACCTGGCAGTTTCTTGGCTTTGTTCG-3’ |
| *nanos* prom rev | 5’-AAGCGTTCTGTTTCGTCACCTCCATCT-3’ |
| 5’FOR | 5’-TCTTGACCTTGCCACAGAGG-3’ |
| 5’REV | 5’-TGACACTTACCGCATTGACA-3’ |
| 3’FORnew | 5’-CATTTGCCTTTCGCCTTATTTTAGA-3’ |
| 3’REVnew | 5’-AAACCTCGATATACTGACCGATAAAAACAC-3’ |
| *attP-*int-fwd | 5’-CCGGCGGCAACCCTCAG-3’ |
| *attP-*int-rev | 5’-ATCGCGCAAATATCTCTTCAAA-3’ |
| *attL*-F-new-2 | 5’-GAGGTCGACGATGTAGGTCAC-3’ |
| *attL*-R-new-2 | 5’-ACCTTTTCTCCCTTGCTACTGAC-3’ |
| *attR*-F-new2 | 5’-TCATTTCCTCCTTAGCATCTTTAT-3’ |
| *attR*-R-new2 | 5’-CTACCGCCACCTCGACCCGTTCAT-3’ |
| *nanos*-int-fwd | 5’-AAACAACAATCAACAACCCGTCCA-3’ |
| *nanos*-int-rev | 5’-AGCCTTTCTTGCCCACCAGTTCC-3’ |
| rpLfwd | 5’-CCCGCTGGCCAAACCGAAAAA-3’ |
| rpLrev | 5’-GTGGGGGAGGAAACGTTGGAATCT-3’ |
| *nanos*intqPCRfwd | 5’-TTCCCGAAAGCAAGATGGATAC-3’ |
| *nanos*intqPCRrev | 5’-CGTTCAGGATGCGTTCGAA-3’ |
| S7qPCRfwd | 5’-ACCACCATCGAACACAAAGTTGAC-3’ |
| newS7qPCRrev | 5’-GCTGCTGCAAACTTCGGCTAT-3’ |
| S7fwd | 5’-GACGCCGCGACCCCAACA-3’ |
| S7rev | 5’-TTTTCTGCGTCCACCCCGATTTCTC-3’ |

Pairs of forward and reverse primers used in PCR and real-time quantitative PCR. Details of reaction conditions can be found in the corresponding section of materials and methods.
